# Impacts of repetitive transcranial magnetic stimulation parameters on cerebral hemodynamics in patients with disorders of consciousness

**DOI:** 10.3389/fneur.2026.1796875

**Published:** 2026-06-10

**Authors:** Hao Peng, Shuai Han, Yilin Zhang, Aoxuan Liu, Fenglin Wu, Mingrui Dong, Siping Wang, Qianqian Ge, Long Xu, Jianghong He, Juanning Si

**Affiliations:** 1 School of Instrumentation Science and Opto-Electronics Engineering, Beijing Information Science and Technology University, Beijing, China; 2 Department of Neurosurgery, Beijing Tiantan Hospital, Capital Medical University, Beijing, China

**Keywords:** disorders of consciousness, functional near-infrared spectroscopy, hemodynamic responses, repetitive transcranial magnetic stimulation, stimulation parameters

## Abstract

**Significance:**

Repetitive transcranial magnetic stimulation (rTMS) has emerged as a promising noninvasive neuromodulation technique for patients with disorders of consciousness (DOC). However, the specific effects of rTMS stimulation parameters on this population remain insufficiently characterized.

**Objectives:**

Functional near-infrared spectroscopy (fNIRS) was used to evaluate the real-time and short-term effects of rTMS with different stimulation parameters on patients with DOC.

**Methods:**

The hemodynamic data were acquired from 20 patients with DOC during rTMS at different frequencies (10 Hz and 20 Hz) and different target sites (at F3 and F4), yielding four experimental conditions. The spatiotemporal characteristics of hemodynamics across these conditions were quantitatively compared to investigate the real-time and short-term after-effects of rTMS.

**Results:**

Results showed that the hemodynamic responses significantly increased following the high- frequency (both 10 Hz and 20 Hz) rTMS. With a fixed total number of stimulation pulses, the lower-frequency, longer-duration protocol (10 Hz, 10 s) produced stronger hemodynamic responses than the higher-frequency, shorter-duration protocol (20 Hz, 5 s) across measured brain areas. Notably, rTMS applied to either the left (F3) or right (F4) DLPFC evoked significantly stronger ipsilateral than contralateral hemodynamic responses, indicating a lateralized neuromodulation effect.

**Conclusion:**

The fNIRS-based monitoring offers a valuable method for evaluating the neuromodulation efficacy of rTMS, providing a novel perspective for optimizing neuromodulation parameters for patients with DOC.

## 1 Introduction

Disorders of consciousness (DOC) are conditions characterized by varying degrees of impairment in wakefulness and awareness following a severe brain injury (1, 2). The development of neurorehabilitation plans is both a major challenge and a promising neuromodulatory direction in clinical neuroscience (3, 4).

In recent years, several neuromodulation techniques have been applied to facilitate consciousness recovery in individuals with severe brain injuries. Transcranial Magnetic Stimulation (TMS) is a non-invasive neuromodulation technique that employs magnetic pulses generated by high-intensity currents flowing through a coil placed on the scalp to alter cortical excitability and modulate neuronal activity of the brain (5, 6). TMS is mainly divided into several modalities: single-pulse TMS (sTMS), paired-pulse stimulation (pTMS), repetitive TMS (rTMS) (7). In patients with DOC, rTMS is primarily used for neuromodulatory intervention. By delivering repeated magnetic pulses, rTMS induces electrical activity in the brain, which modulates cortical excitability and thus achieves the goal of neuromodulation (3). Multiple studies have shown that high-frequency rTMS can improve the CRS-R scores in patients with DOC (8, 9). Furthermore, a meta-analysis involving 90 patients with DOC also indicated that rTMS may have a positive effect on promoting consciousness recovery (10). These findings collectively highlight the value of rTMS in the field of DOC.

However, the specific design of rTMS parameters (frequency, target, duration, pulse number, intensity, coil and other factors) significantly influences the stimulation outcomes in patients with DOC (11, 12). Previous studies that monitored cortical oxygenation changes induced by rTMS have found that low-frequency (1 Hz) rTMS significantly reduces oxygenated hemoglobin (HbO) levels in specific brain regions, whereas high-frequency (≥5 Hz) rTMS significantly increases them and induces long-term potentiation-like plasticity (13). The design of rTMS target is also crucial for promoting consciousness recovery in patients with DOC, as different sites induce distinct clinical and physiological changes. Prior studies have targeted regions such as the frontal lobe (14), motor cortex, and the angular gyrus (AG). Although current evidence provides no clear indication of the optimal stimulation target for patients with DOC, the DLPFC remains the most frequently selected target in clinical rTMS applications (15). Stimulating the DLPFC with rTMS enhanced neural activity in the prefrontal cortex and subsequently modulated functional connectivity within the DMN, potentially exerting a positive influence on consciousness recovery in patients with DOC (16). However, the spatiotemporal characteristics of hemodynamics of different rTMS parameters remain unclear. Consequently, there is an urgent need to develop a rapid, quantitative approach that can be used to evaluate the spatiotemporal responsiveness differences in hemodynamics across varying rTMS parameters.

In recent years, functional near-infrared spectroscopy (fNIRS), as an emerging non-invasive optical imaging technology, has attracted growing attention in the detection of residual awareness, assessment of neuromodulation effects, and exploration of the underlying mechanism of DOC (17, 18). Compared to functional magnetic resonance imaging (fMRI), fNIRS offers superior temporal resolution, portability, and favorable scalability. Unlike electroencephalography (EEG), fNIRS enables higher spatial precision for localizing and analyzing specific cortical regions. More importantly, fNIRS is not subject to interference from electrical stimulation and is less susceptible to motion artifacts. These advantages collectively make it suitable for investigating the real-time spatiotemporal responsiveness of brain activity in patients with DOC during the neuromodulation period. In recent years, fNIRS has been used to assess brain activity changes in response to various neuromodulation therapies, including spinal cord stimulation (SCS) (19), and deep brain stimulation (DBS).

Notably, fNIRS has been increasingly applied to evaluate the effects of rTMS in various cognitive disorders (such as Alzheimer’s disease and aphasia) (20) and neuropsychiatric conditions (including schizophrenia and phobia) (21). However, studies using fNIRS to investigate the neuromodulation effects of rTMS on patients with DOC are still fragmented and limited. Our previous work utilized fNIRS to investigate the influence of rTMS effects on hemodynamic responses for patients with DOC. The results showed that the distribution patterns of the hemodynamic responses following rTMS varied during multi-frequency rTMS applied to the F3 region, with 10 Hz eliciting strong frontal cortex responses and 20 Hz inducing larger ones in the motor-related cortex, pointing to a potential positive effect of high-frequency rTMS on consciousness recovery (22). Nevertheless, certain issues still need to be explored. On one hand, the spatiotemporal responses elicited by different rTMS parameters, such as stimulation target and duration, have not been systematically compared or elucidated. On the other hand, the underlying mechanisms of rTMS-mediated neuromodulation still require further clarification.

In view of this, the current study further investigates the effects of rTMS with different stimulation parameters on patients with DOC. The main objectives of this study were threefold: (1) to investigate the real-time and short-term distribution of hemodynamic responses evoked by rTMS; (2) to compare the differences in spatiotemporal characteristics of hemodynamic responses across distinct rTMS conditions and to identify the effective rTMS protocol; (3) to explore the potential mechanisms of rTMS in facilitating the recovery of consciousness in patients with DOC.

## 2 Materials and methods

### 2.1 Participants

Twenty subjects (11 males and 9 females, age: 49.30 ± 14.59 years, mean CRS-R score: 8.20 ± 3.66) from the Department of Neurosurgery in Beijing Tiantan Hospital and Fengtai Rehabilitation Hospital of Beijing Municipality (Tieying Hospital) were recruited. The inclusion criteria of the subjects were as follows: (1): aged between 18 and 65 years; (2) diagnosed with unresponsive wakefulness syndrome (UWS) or minimally conscious state (MCS) based on the CRS-R scale; The CRS-R consists of six subscales evaluating auditory, visual, motor, verbal, communication, and arousal functions, with a total score ranging from 0 to 23 (23). The clinical diagnostic criteria for MCS and UWS should comply with the following standards. For MCS: auditory (3–4), visual (2–5), motor (3–5), verbal (3), communication (1), and arousal (≤ 2). For UWS: auditory (≤ 2), visual (≤ 1), motor (≤ 2), verbal (≤ 2), communication (0), and arousal (≤ 2). (3) Etiology including traumatic brain injury (TBI), intracerebral hemorrhage (ICH), or hypoxic–ischemic encephalopathy (HIE), with a disease duration of more than 28 days. Written informed consent was obtained from the patients’ caregivers. The experimental protocol was approved by the ethics committees of the Beijing Tiantan Hospital (approval number: KY2022-094-02). The clinical characteristics of the patients with DOC are illustrated in Table 1.

**Table 1:** Clinical data of the patients with disorders of consciousness.

No.	Age/Gender	Diagnosis	Duration of DOC (months)	Etiology	CRS-R
1	60/M	UWS	5.3	TBI	3 (002100)
2	38/F	UWS	4.3	ICH	7 (112102)
3	65/F	UWS	7.0	ICH	6 (102102)
4	29/M	UWS	2.3	ICH	6 (111102)
5	63/M	UWS	10.4	ICH	5 (002102)
6	47/M	UWS	4.0	HIE	6 (102102)
7	50/M	UWS	7.6	HIE	6 (102102)
8	65/F	UWS	6.8	HIE	6 (102102)
9	52/M	UWS	3.0	HIE	5 (002102)
10	31/M	MCS	4.1	TBI	10 (322102)
11	19/M	MCS	5.8	TBI	8 (131102)
12	59/F	MCS	8.9	TBI	9 (024102)
13	58/F	MCS	7.0	ICH	9 (102213)
14	59/F	MCS	10.6	ICH	10 (133102)
15	58/F	MCS	3.5	TBI	20 (456320)
16	54/F	MCS	10.8	ICH	8 (132002)
17	63/F	MCS	5.7	ICH	9 (132102)
18	41/M	MCS	3.3	TBI	14 (334202)
19	54/M	MCS	7.3	HIE	8 (113102)
20	21/M	MCS	12.5	HIE	9 (132102)

CRS-R, coma recovery scale-revised; UWS, unresponsive wakefulness syndrome; MCS, minimally conscious state; TBI, traumatic brain injury; ICH, intracerebral hemorrhage; HIE, hypoxic–ischemic encephalopathy.

### 2.2 Study design

The experimental paradigm was designed based on the study by Bai et al. (24) and our previous work (22). Using the NS3000 system (YIRUIDE Medical Co., Wuhan, China) system, rTMS was conducted at two different stimulation frequencies (10 Hz and 20 Hz) and two different stimulation targets: left (F3) and right (F4) of the dorsolateral prefrontal cortex. The stimulation intensity was set at 100% of the resting motor threshold (RMT) (25). In accordance with the recommendations issued by the International Federation of Clinical Neurophysiology (IFCN) Committee, the RMT was defined as the minimum stimulus intensity capable of evoking motor evoked potentials (MEPs) exceeding 50 μV in 5 out of 10 trials of the relaxed abductor pollicis brevis muscle (26).

**Figure 1:**
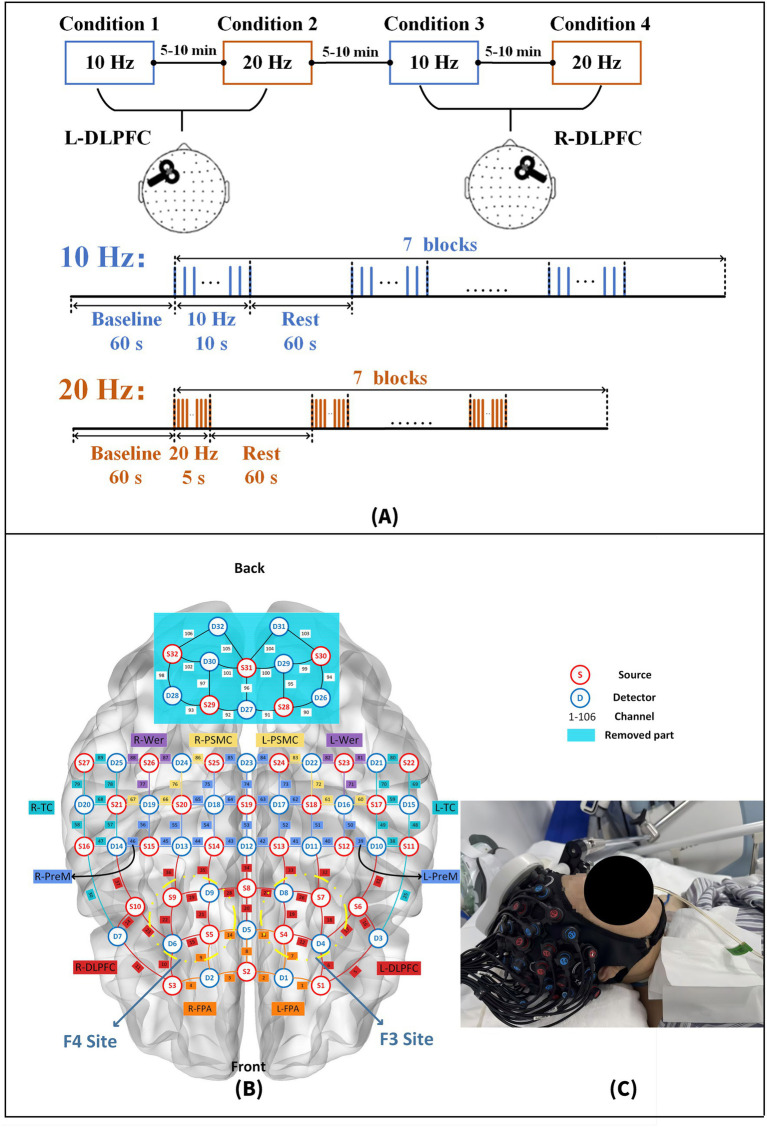
Study protocol. **(A)** Experimental paradigm for rTMS. **(B)** Arrangement of fNIRS optodes on the head. **(C)** Photograph of the experimental setup.

A blocked-design protocol was used in this study. There were 4 rTMS conditions: condition 1 (target: F3, frequency: 10 Hz, duration: 10 s); condition 2 (target: F3, frequency: 20 Hz, duration: 5 s); condition 3 (target: F4, frequency: 10 Hz, duration: 10 s); condition 4 (target: F4, frequency: 20 Hz, duration: 5 s). Each rTMS condition consisted of 7 blocks, with each block consisting of an rTMS stimulation period followed by a 60 s rest period. The total number of stimulation pulses was fixed at 700 across the 4 conditions. The experiment was conducted pseudo-randomly. A 5–10 min interval separated the four conditions to mitigate residual effects of the prior session. To ensure a balance between data reliability and patient comfort, the practical interval was constrained by the duration of the essential quality control calibration procedure, resulting in an actual average interval ranging from 5 to 10 min. rTMS target positioning followed the International 10–20 EEG system (Figure 1). The TMS coil was positioned under the guidance of a clinical specialist and stabilized using a built-in robotic arm.

### 2.3 Data acquisition

In this study, the hemodynamic data were acquired using the BS-7000S (YIRUIDE Medical Co., Wuhan, China) system, which utilizes light wavelengths of 690 nm and 830 nm to quantify concentration changes in concentrations of oxygenated (HbO) and deoxygenated (HbR) hemoglobin in the cerebral cortex. Thirty-two light sources (red circles) and 32 detectors (blue circles) were placed over the brain, yielding a total of 106 optical channels. The arrangement of the optodes and the parcellation of the 12 regions of interest (ROIs) are shown in Figure 1 and Table 2. The distance between the light source and the detector pairs was 3 cm. The sampling rate of the fNIRS system was 20 Hz.

**Table 2:** The arrangement of fNIRS optical channels on different brain regions.

No.	ROIs	Channels
1	Left frontal polar area (L-FPA)	1, 2, 7, 8,13
2	Right frontal polar area (R-FPA)	3, 4, 9, 14
3	Left dorsolateral prefrontal cortex (L-DLPFC)	5, 6, 12, 16, 17, 18, 19, 20, 26, 27, 31, 32, 33, 34
4	Right dorsolateral prefrontal cortex (R-DLPFC)	10, 11, 15, 21, 22, 23, 24, 28, 29, 35, 36, 37
5	Left pre-motor cortex (L-PreM)	39, 40, 41, 42, 50, 51, 52, 53, 62, 63, 73, 74, 84
6	Right pre-motor cortex (R-PreM)	43, 44, 45, 46, 54, 55, 56, 64, 65, 75, 85
7	Left primary sensorimotor cortex (L-PSMC)	60, 61, 72, 83
8	Right primary sensorimotor cortex (R-PSMC)	66, 67, 76, 86
9	Left Wernicke area (L-Wer)	71, 81, 82
10	Right Wernicke area (R-Wer)	77, 87, 88
11	Left temporal cortex (L-TC)	25, 38, 48, 49, 59, 69, 70, 80
12	Right temporal cortex (R-TC)	30, 47, 57, 58, 68, 78, 79, 89

Considering the condition of patients with DOC, data acquisition was performed in the supine position. To optimize data quality and patient comfort, all occipital channels were excluded. To prevent potential electromagnetic interference from the TMS coil with the fNIRS optodes, the corresponding light sources and detectors on the fNIRS cap were temporarily removed during stimulation. Specifically, when stimulating the F3 site, sources (S4, S7) and detectors (D4, D8) were removed; correspondingly, when stimulating the F4 site, sources (S5, S9) and detectors (D6, D9) were removed. The signals from these optical channels were excluded from subsequent data preprocessing.

### 2.4 Data analysis

In this study, data preprocessing was performed using HOMER 2, and subsequent analysis was conducted in MATLAB 2023a (MathWorks, Natick, MA, United States). Firstly, the signal-to-noise ratio was evaluated by calculating the coefficient of variation (CV) for each channel (27). Channels with a CV greater than 15% were excluded. This step removed channels invalidated by source/detector removal. Next, raw light intensity signals were converted into optical density (OD) signals based on the modified Beer–Lambert Law (28). Principal component analysis (PCA) was applied to remove the systemic physiological interference (nSV = 0.8) (29). Subsequently, channel-by-channel automatic inspection of motion artifacts was performed. Spline interpolation was utilized to correct and reconstruct the identified motion artifact segments. Following this, a band-pass filter (0.01–0.1 Hz) was applied to remove task-irrelevant physiological noise such as heartbeat (approximately 1.1 Hz), breathing (around 0.3 Hz) and blood pressure fluctuations (30). Afterward, the optical density signals were converted into hemoglobin concentration (Conc) signals. Differential pathlength factor (DPF) was calculated for each individual (31). Finally, the data were segmented into epochs from −5 s to 35 s. For quantitative analysis, 12 distinct regions of interest (ROIs) were defined across the cerebral cortex, covering the bilateral frontopolar, dorsolateral prefrontal, pre-motor, primary sensorimotor, Wernicke, and temporal cortices. The arrangement of fNIRS optical channels across different brain regions is illustrated in Table 2. Similar to our prior work (22), the representative channel for each brain region was determined by comparing the hemoglobin signals during stimulation period (0–10 s for 10 Hz; 0–5 s for 20 Hz) to the baseline period (−5–0 s) and selecting the channel with the highest t-value. On this basis, the area under the curve (AUC) of the hemodynamic responses for each subject during three periods of stimulus (P1: 0–10 s, P2: 10–20 s, P3: 20–30 s) was extracted for feature quantification.

For statistical analysis, SPSS 27.0 (IBM Corporation, New York, United States) and GraphPad Prism 10 (GraphPad Software Inc., San Diego, United States) were employed to perform two-sample t-test and analysis of variance (ANOVA). For the two-sample t-test, false discovery rate (FDR) correction was applied for multiple comparisons, and Cohen’s d was used to estimate the corresponding effect size. For ANOVA, the least significant difference (LSD) method was employed for *post hoc* pairwise comparisons, while partial *η*^2^ was adopted to quantify the effect size. The results were visualized with the BrainNet Viewer (32).^1^ Unless otherwise mentioned, the results are presented as the mean ± standard error (SE). The differences were accepted as significant when *p* < 0.05.

## 3 Results

### 3.1 Real-time effects of hemodynamic responses under rTMS frequencies on bilateral brain regions

**Figure 2:**
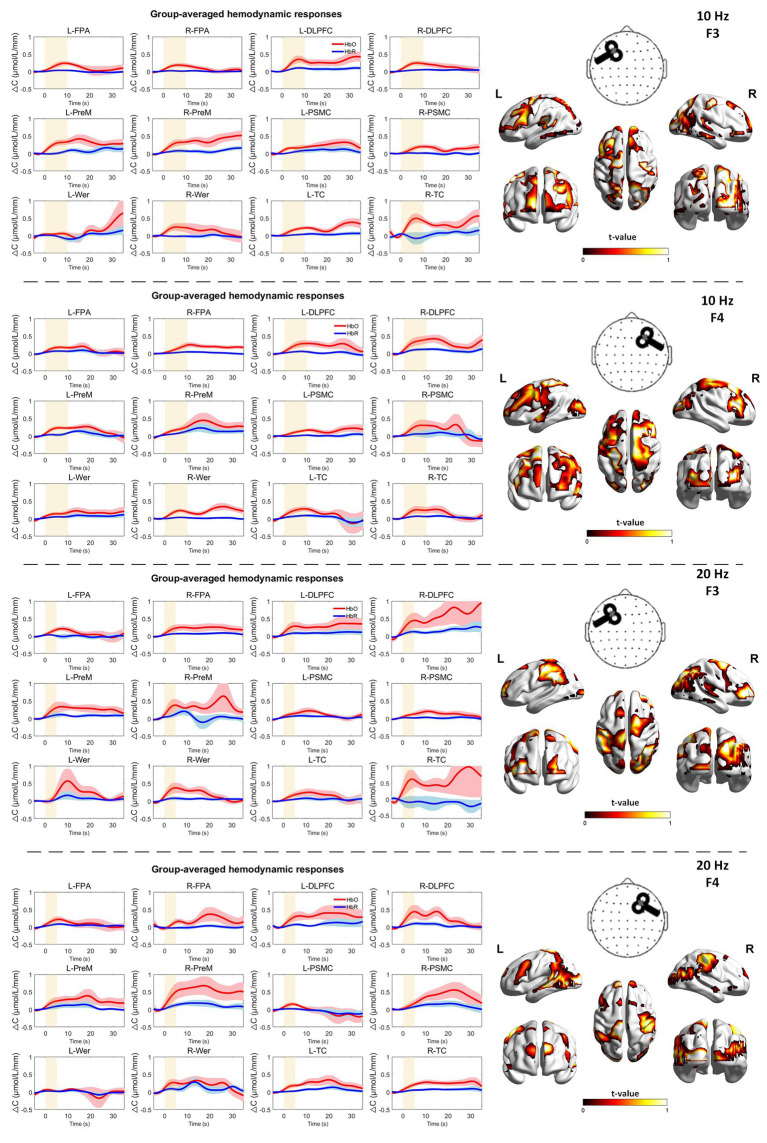
Real-time effects of hemodynamic responses and cortical activation induced by 10/20 Hz rTMS over the F3/F4.

Hemodynamic responses elicited by rTMS at different frequencies show substantial variations when applied to bilateral brain regions. Specifically, high-frequency rTMS targeting either the F3 or F4 region induced a significant increase in HbO concentration across most cerebral areas. The group-averaged hemodynamic responses evoked by different rTMS frequencies are contrasted in Figure 2, which also presents the corresponding activation patterns observed during stimulation. Upon rTMS onset, the spatiotemporal characteristics of hemodynamic responses under rTMS with different parameters were different. During the rTMS periods, significant increases in hemodynamic responses were observed in the ROIs compared to the baseline (−5–0 s). The increase was observed in the bilateral FPA, DLPFC, PreM, PSMC, Wer, and TC, with varying magnitudes across these regions.

### 3.2 The impact of rTMS parameter variations on hemodynamic responses during bilateral brain stimulation

**Figure 3:**
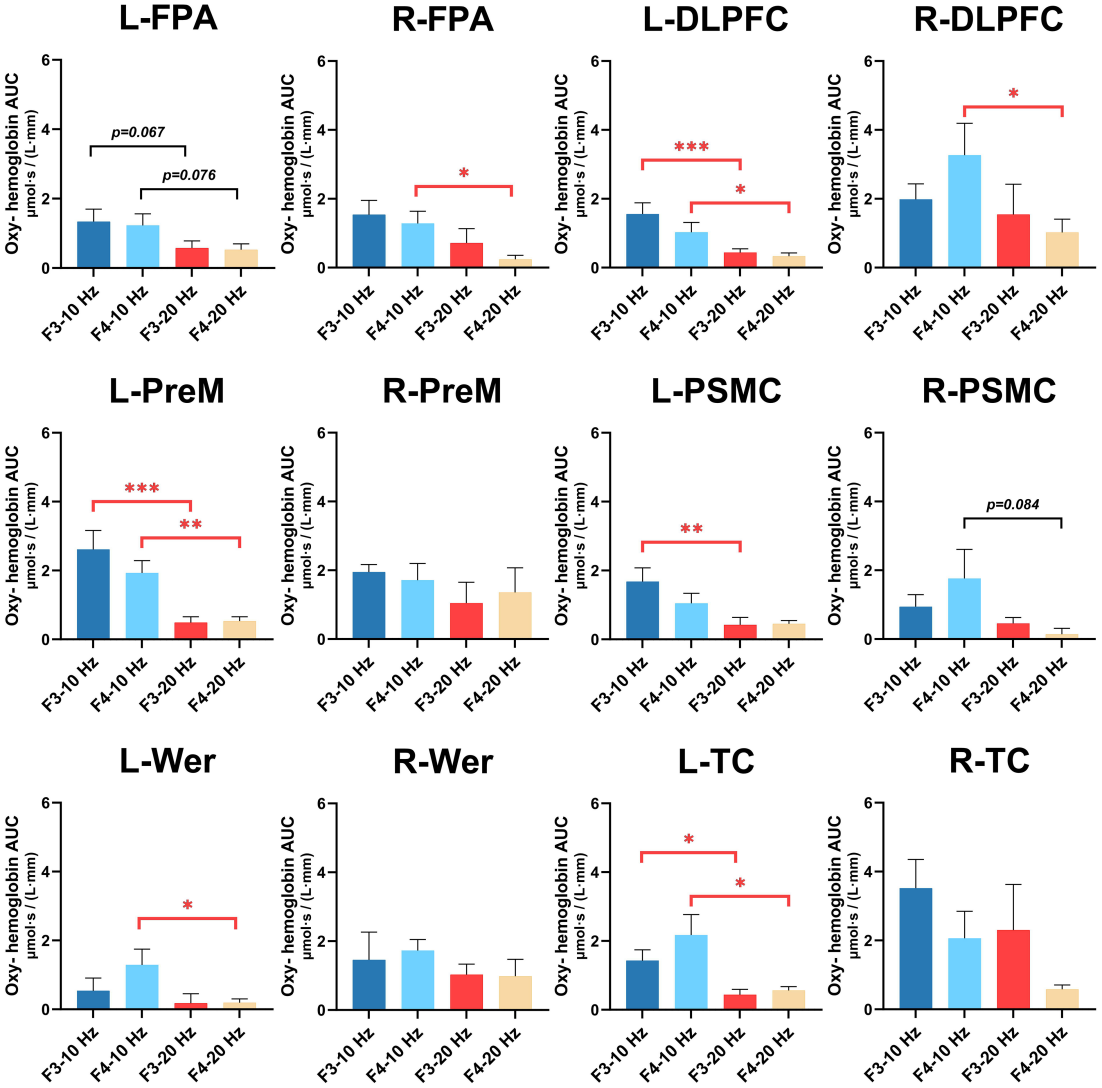
Comparison of changes in the AUC of hemodynamic responses induced by rTMS parameters.

As illustrated in Figure 3, ANOVA was performed to investigate different neuromodulatory effects of rTMS parameters. For the right frontal polar area, the AUC values during stimulation [*F* (3, 68) = 2.966, *p* = 0.0381, Partial *η*^2^ = 0.116] indicated significant differences. Specifically, when stimulating the F4 region, 10 Hz rTMS elicited greater hemodynamic responses compared to 20 Hz (*p* = 0.007, Cohen’s d = 0.981).

Results from the L-DLPFC indicated that rTMS parameters significantly influenced the hemodynamic responses [*F* (3, 71) = 6.124, *p* < 0.001, Partial *η*^2^ = 0.206]. 10 Hz TMS elicited significantly greater changes in HbO concentration compared to 20 Hz rTMS (*p* = 0.002, Cohen’s d = 1.101 for F3 area; p = 0.031, Cohen’s d = 0.760 for F4 area). Otherwise, no significant differences were observed in the R-DLPFC. Notably, stimulation at the F4 region elicited stronger hemodynamic responses at 10 Hz compared to 20 Hz rTMS (*p* = 0.038, Cohen’s d = 0.738).

For the left pre-motor cortex, a group-level analysis revealed a significant main effect [*F* (3, 71) = 9.085, *p* < 0.001, Partial *η*^2^ = 0.277]. Specifically, when stimulating the F3 site, 10 Hz rTMS elicited stronger hemodynamic responses during the stimulation period compared to 20 Hz rTMS (*p* < 0.001, Cohen’s d = 1.230). Consistently, 10 Hz stimulation produced greater hemodynamic responses than 20 Hz at the F4 target site (*p* < 0.001, Cohen’s d = 1.205).

In the primary sensorimotor cortex, group-average analysis of the left hemisphere revealed a significant difference in the hemodynamic responses [*F* (3, 62) = 4.754, *p* = 0.005, Partial *η*^2^ = 0.187]. Specifically, 10 Hz rTMS applied to the F3 site elicited significantly stronger cortical hemodynamic responses compared to 20 Hz rTMS (*p* = 0.011, Cohen’s d = 1.005).

In the left Wernicke area, when stimulating the F4 site, 10 Hz rTMS induced a greater increase in HbO concentration than 20 Hz stimulation (*p* = 0.047, Cohen’s d = 0.801).

Notably, the left temporal cortex also exhibited significant differences in the AUC values of hemodynamic responses at the group level [*F* (3, 69) = 5.014, *p* = 0.003, Partial *η*^2^ = 0.179]. Further analysis indicated that when stimulating the F3 site with rTMS, the 10 Hz condition produced a significantly greater increase in HbO concentration than the 20 Hz condition (*p* = 0.010, Cohen’s d = 0.931). This effect was consistent during stimulation of the F4 site, where the 10 Hz protocol also elicited stronger responses compared to 20 Hz (*p* = 0.010, Cohen’s d = 0.875).

### 3.3 Short-term aftereffects of rTMS stimulation

**Figure 4:**
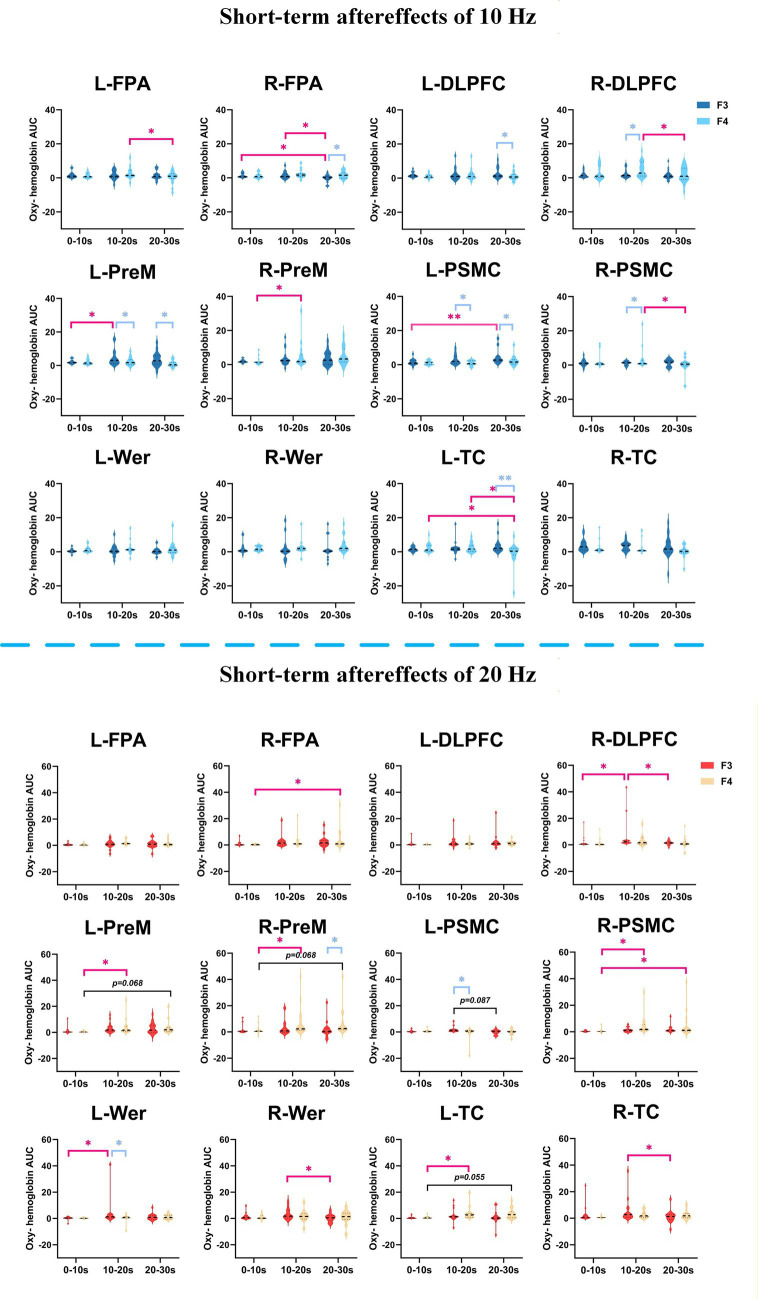
Short-term aftereffects of hemodynamic responses following 10/20 Hz rTMS over the F3/F4 regions.

The HbO concentration changes within 3 periods after stimulus onset were extracted, and the AUC for each period was calculated as a quantitative feature. Figure 4 displayed a comparison of hemodynamic responses AUC values across 3 time-windows under 10 and 20 Hz rTMS, as analyzed using ANOVA. Under 10 Hz rTMS applied to the F3 area, differences in short-term aftereffects were observed across the R-FPA, L-PreM, and L-PSMC cortices. Specifically, group-level analysis of the R-FPA revealed a significant main effect [*F* (2, 46) = 4.879, *p* = 0.012, Partial *η*^2^ = 0.175], the hemodynamic responses during both the P1 (0–10 s) period (*p* = 0.041, Cohen’s d = 0.966) and P2 (10–20 s) period (*p* = 0.023, Cohen’s d = 0.864) were significantly higher than those during the P3 (20–30 s) period. In the L-PreM, the responses during the P2 (10–20 s) period were significantly stronger compared to the P1 (0–10 s) period (*p* = 0.049, Cohen’s d = 0.688). Additionally, in the L-PSMC, the group-level analysis revealed a significant main effect of period [*F* (2, 39) = 3.119, *p* = 0.05, Partial *η*^2^ = 0.138], with hemodynamic responses during the P3 (20–30 s) period being significantly greater than that during the P1 (0–10 s) period after stimulus onset (*p* = 0.009, Cohen’s d = 0.918).

Interestingly, when the same frequency (10 Hz rTMS) was applied to the F4 area, short-term aftereffects were detected in the corresponding symmetric brain regions mentioned. Specifically, in the L-FPA, the hemodynamic response during the P2 (10–20 s) period was significantly higher than that during the P3 (20–30 s) period (p = 0.045, Cohen’s d = 0.632). Similarly, in the R-PreM, the responses in the P2 (10–20 s) period were significantly stronger compared to the P1 (0–10 s) period (*p* = 0.041, Cohen’s d = 0.685). Finally, for the R-PSMC, HbO concentration during the P2 (10–20 s) period was significantly greater than that during the P3 (20–30 s) period (*p* = 0.035, Cohen’s d = 0.625).

Compared the effects of stimulation sites on hemodynamic responses within the same time period, we found that 10 Hz rTMS induced an ipsilateral dominance effect in HbO changes. Specifically, stimulation of the F3 site elicited significantly stronger HbO changes in the left hemisphere regions including L-PreM (*p* = 0.036, Cohen’s d = 0562 in P2 (10–20 s) period; *p* = 0.013, Cohen’s d = 0.764 in P3 (20–30 s) period), L-PSMC (*p* = 0.049, Cohen’s d = 0.412 in P2 (10–20 s) period; *p* = 0.023, Cohen’s d = 0.528 in P2 (10–20 s) period), and L-TC (*p* = 0.001, Cohen’s d = 0.760 in P3 (20–30 s) period) compared to the effects induced by F4 site. Conversely, stimulation of the F4 site induced a greater enhancement of HbO in the right hemisphere regions including R-FPA (*p* = 0.003, Cohen’s d = 0.956 in P3 (20–30 s) period), R-DLPFC (*p* = 0.039, Cohen’s d = 0.653 in P2 (10–20 s) period), and R-PSMC (*p* = 0.048, Cohen’s d = 0.580 in P2 (10–20 s) period) than that achieved by stimulating the F3 site.

As shown in the bottom part of Figure 4, the graph illustrated the short-term effects of 20 Hz rTMS on different brain regions, the ipsilateral dominance effect observed too. Specifically, in the L-PSMC (*p* = 0.011, Cohen’s d = 0.599 in P2 (10–20 s) period) and L-Wer (*p* = 0.031, Cohen’s d = 0.474 in P2 (10–20 s) period), stimulation at F3 elicited significantly stronger hemodynamic responses compared to F4. In contrast, for the R-PreM (*p* = 0.042, Cohen’s d = 0.607 in P3 (20–30 s) period), stimulation at F4 resulted in significantly greater responses than stimulation at F3.

## 4 Discussion

### 4.1 The real-time and short-term effects of rTMS stimulation frequency and target on hemodynamics

In this study, fNIRS was utilized to investigate the distribution of hemodynamic responses of rTMS with different stimulation parameters for patients with DOC. Viewed in terms of real-time effects, the results demonstrated that 10 Hz rTMS stimulation over the F3 area significantly increased bilateral HbO concentrations in the FPA, PreM, PSMC, Wer, and TC regions, suggesting that rTMS of the DLPFC can enhance functional brain connectivity (22). Furthermore, when stimulating the F4 area, significant enhancements in hemodynamic responses were also observed, which was consistent with the results reported by Naro et al. Their study applied 10 Hz rTMS over the right DLPFC in patients with DOC and found partial restoration of brain connectivity and transient enhancement of consciousness (35). It’s worth noting that the DLPFC plays a critical role in higher cognitive functions (36). Considering the short-term aftereffects, the results showed that under 10 Hz stimulation of the F3 site, significant short-term aftereffects were observed in the R-FPA, L-PreM, and L-PSMC regions. Notably, when stimulating the F4 site, significant short-term aftereffects were also detected in the contralateral symmetric regions including L-FPA, R-PreM, and R-PSMC.

Similarly, viewed in terms of real-time effects, 20 Hz rTMS also induced a significant increase in HbO concentration. The short-term aftereffects of 20 Hz rTMS show regional specificity between the F3 and F4 sites. Previous researches have demonstrated that applying 20 Hz rTMS to the F3 or F4 regions in patients with DOC led to positive outcomes across multiple metrics, including the CRS-R, subscales of the disorders of consciousness scale (DOCS), and electroencephalography (EEG), suggesting a definitive neuromodulatory effect on promoting consciousness recovery (37).

### 4.2 Comparison of the rTMS effects under different conditions

This study quantitatively analyzed hemodynamic changes under four conditions combining two stimulation frequencies and two cortical targets. The results demonstrated that, with the total number of pulses held constant, lower-frequency and longer-duration protocol (10 Hz, 10 s) produced stronger hemodynamic responses than the higher-frequency and shorter-duration protocol (20 Hz, 5 s) across the brain areas. We proposed the hypothesis that the neuromodulatory effects of high-frequency rTMS build up with prolonged stimulation. Previous studies suggested that prolonging the duration of rTMS stimulation could extend the duration of cognitive effects (38). Clinical evidence in depression showed that extended treatment duration was associated with additional clinical benefits. Furthermore, investigations in neuropathic pain have revealed that insufficient stimulation duration or an inadequate number of sessions led to diminished efficacy and reduced persistence of the therapeutic effect (39). Furthermore, evidence from analyses based on motor evoked potential amplitudes, spectral power density, and functional connectivity indicated that high-frequency rTMS applied with different durations produced distinct neuromodulatory outcomes (40). However, other studies argued that within the high-frequency range (5–20 Hz), the relationship among stimulation frequency, duration, and clinical efficacy did not follow simple linear patterns (34).

Apart from stimulation duration, we propose that high-frequency rTMS applied bilaterally over the DLPFC may exhibit an ipsilateral dominance effect. The DLPFC is frequently selected as a target due to its crucial role in cognitive functions such as learning, memory, which are closely linked to consciousness processing. Some studies have indicated that stimulating the left versus right DLPFC may lead to distinct clinical outcomes, which is consistent with the results of our investigation. Moreover, the left DLPFC is primarily involved in analytic processing of stimulus information, plays a critical role in memory encoding, and mediates cognitive control through top-down regulatory mechanisms that suppress inappropriate responses (41). In contrast, the right DLPFC is more engaged in bottom-up attentional control mechanisms (42). Other studies suggested that high-frequency rTMS may enhance excitability in the stimulated cortex through interhemispheric inhibition mechanisms, while simultaneously suppressing activity in contralateral homologous regions (43).

### 4.3 Underlying neural mechanisms of rTMS in the recovery of consciousness

To appreciate the potential neuromodulatory mechanisms of rTMS, it is instructive to first consider the pivotal role of its primary stimulation target DLPFC. In fact, the DLPFC is involved not only involved in higher-order cognition, motor control, and decision-making but also serves as a key hub of the default mode network (DMN). Disruption of functional networks such as the DMN may underlie disorders of consciousness, leading to impaired ability to process internal and external stimuli (44). Studies have shown that rTMS targeting the DLPFC can modulate DMN connectivity (45). Given that the impairment of DMN functional connectivity is closely related to the severity of consciousness disorders, and its restoration aligns with improvements in clinical consciousness levels, however, the neuromodulatory effects produced by stimulating different rTMS sites vary significantly. For instance, in the treatment of depression, high-frequency rTMS over the left and right DLPFC produced different neuromodulatory effects, even with identical stimulation parameters (46). In studies on obsessive-compulsive disorder, rTMS targeting the right DLPFC has been shown to effectively enhance activation levels in this brain region (33). Conversely, in the treatment of attention-deficit/hyperactivity disorder, stimulating the left DLPFC has been found to alleviate hyperactive and impulsive symptoms (47).

These site-dependent differences in effects are likely attributable to the functional lateralization of the cerebral hemispheres: the left DLPFC is primarily responsible for encoding specific details in episodic memory, thereby playing a critical role in memory formation (41), whereas changes in the right DLPFC are closely associated with improvements in attention and memory functions. Given its central role in the mesocircuit model, the DLPFC is considered an ideal target for rTMS. The model posits that extensive neuronal damage leads to deafferentation of thalamocortical and corticostriatal axonal terminals, resulting in reduced striatal activity. This suppression diminishes facilitatory input to the thalamus, thereby weakening thalamocortical drive. As a consequence, thalamocortical connectivity is impaired and overall cortical activation declines (48). The frontocortico-striatopallidal-thalamocortical circuit forms a closed-loop system; disruption at any node within this network can lead to selective impairments in frontal lobe-mediated executive, cognitive, and motivational functions (49). By stimulating the prefrontal or parietal cortex, rTMS may theoretically enhance prefrontal-thalamic functional connectivity through the reestablishment of cortico-subcortical pathways (48). The indirect modulation of the striatum can disinhibit the thalamus, thereby strengthening thalamocortical connections. Furthermore, rTMS-mediated effects may involve several potential mechanisms, including the promotion of synaptic plasticity to enhance functional connectivity and elevated neurotransmitter release in mesostriatal, mesolimbic, and striatal regions, along with increased brain-derived neurotrophic factor (BDNF) expression for the modulation of neural circuits and brain networks (50).

### 4.4 Limitation and further considerations

This study has certain limitations. Firstly, the sample size is relatively limited and the etiologies are varied among the patients with DOC. In further studies, future enrollment criteria should be more strictly controlled, individual differences in etiology and brain lesion location should be considered, thereby enabling a more comprehensive assessment of the effects of rTMS on patients with DOC. Secondly, the current study primarily focused on the investigation of different stimulation targets and stimulation frequencies on hemodynamics in patients with DOC. Other rTMS parameters, including asymmetric stimulation sites (e.g., DLPFC, M1, posterior parietal cortex), stimulation intensity, pulse width, and inter-train interval, may exert unique effects on neuromodulation outcomes and warrant further investigation. Meanwhile, the design of sham control conditions is also a noteworthy consideration, which could further improve the rigor and causal interpretability of the research results. Furthermore, this study mainly analyzed the short-term effects induced by rTMS, while its long-term effects remain to be verified in further research. Long-term follow-up studies are warranted to explore the long-term neuromodulatory value of different stimulation parameters in patients with DOC.

## Data Availability

The raw data supporting the conclusions of this article will be made available by the authors, without undue reservation.

## Footnotes

1
http://www.nitrc.org/projects/bnv/
